# Supplementary material for: Efficient cell-free translation from diverse human cell types
Source: J Biol Chem. 2025 May 28;301(7):110307. doi: 10.1016/j.jbc.2025.110307 (PMC12226075; doi:10.1016/j.jbc.2025.110307)
Supplement: Supporting Information [file mmc1.pdf]

Supplementary figures

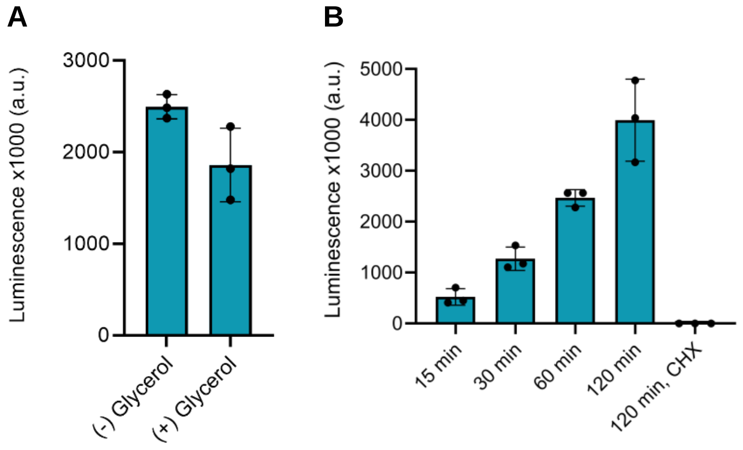

Supplementary Figure 1| Characterization of HeLa S3 lysate
(A) Renilla luciferase assay comparing the translation efficiency of HeLa S3 lysate prepared with or without 15% glycerol in the translation buffer. **(B)** Renilla luciferase assay of time-course *in vitro* translation reactions of HeLa S3 lysate. For (A) and (B) *in vitro* translation reactions contained 5 fmol 3xFLAG-RLuc mRNA/ µl and were performed at 37°C for 1h with a lysate concentration of $1x10^5$ cell equivalent /ml in a total volume of 25 µl of which everything was used for the Renilla Luciferase Assay. Each dot depicts the value of an individual experiment for which the luminescence was measured three times. Mean and SD are shown.

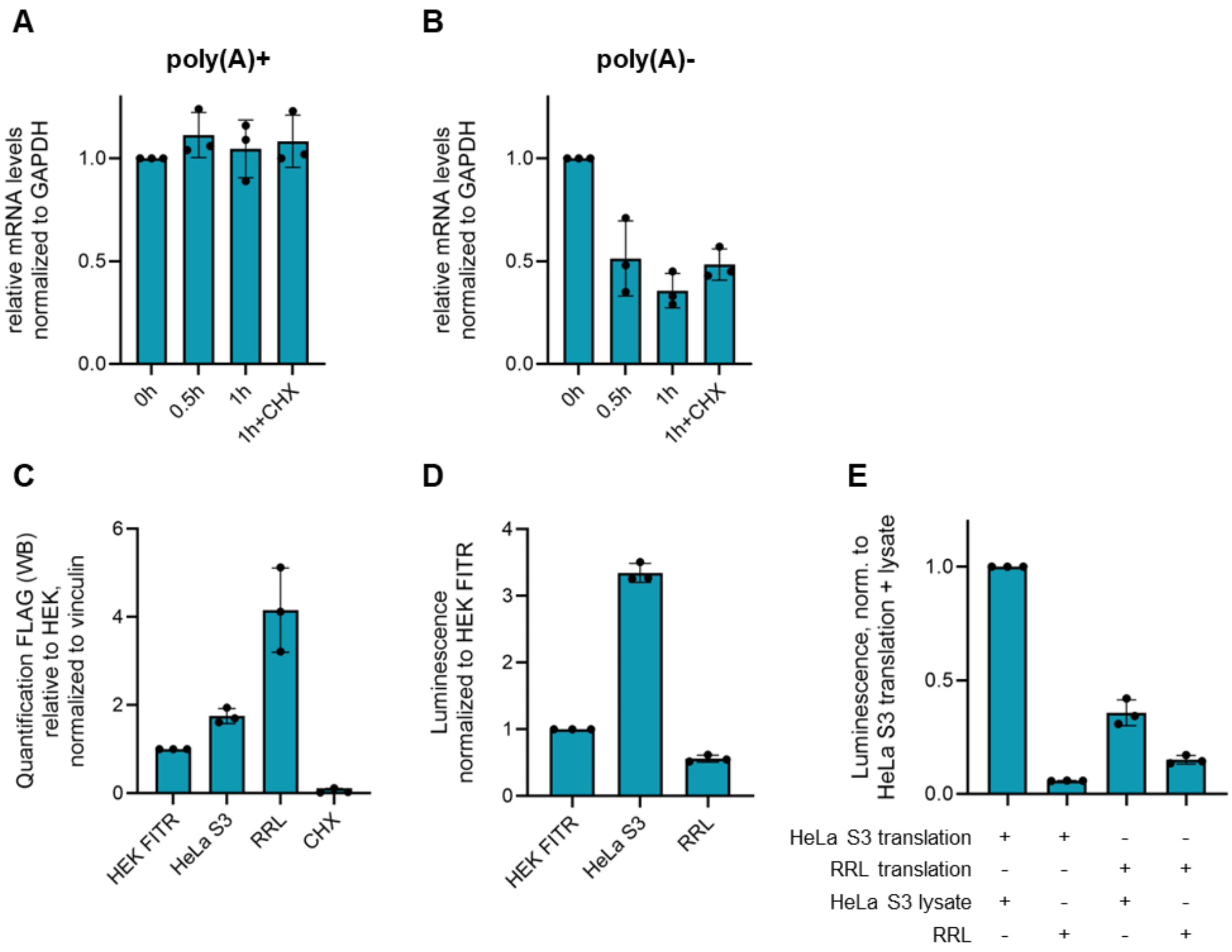

Supplementary Figure 2| Characterization of HEK FITR lysate

(A-B) Relative levels of 3xFLAG-HBB reporter mRNAs with (A) and without (B) a poly(A) tail after *in vitro* translation for 0 to 1 hour in HEK FITR lysate assessed by RT-qPCR. Ct-values are normalized to GAPDH and the respective 0-hour samples. **(C-D)** *In vitro* translation reactions of the 3xFLAG-RLuc reporter mRNA performed in HEK FITR, HeLa S3, or rabbit reticulocyte lysate (RRL), analysed by protein synthesis quantification based on band intensity in the anti-FLAG-stained blot (Figure 2I) (C) or Renilla luciferase assay (D). RRL translation reactions were performed according to the manufacturer's instructions, using a lysate concentration of 70% and an incubation temperature of 30 °C. HeLa S3 and HEK FITR reactions were adjusted to a lysate concentration of 1.4 × 10^5 cell equivalents/µL and were incubated at 37 °C. **(E)** Renilla luciferase activity of HeLa S3 and RRL *in vitro* translation reactions mixed 1:1 either with HeLa S3 lysate or RRL before measuring the luminescence. All translation reactions were performed with 5 fmol RNA/µL and incubated for 1 hour at 37°C, unless otherwise stated. For (A, B) 80 µL translation reactions were prepared of which 4 µL were used for western blot analysis (Fig. G) and the rest was used for RNA isolation for subsequent RT-qPCR analysis. For (C-E) 50 µL translation reactions were prepared of which 2 µL were used for western blot analysis (Fig. 2I), and 25 µL and 12.5 µL were used for luciferase analysis for (D) and (E), respectively. (A-E) Each dot depicts the value of an individual experiment. Mean and SD are shown.

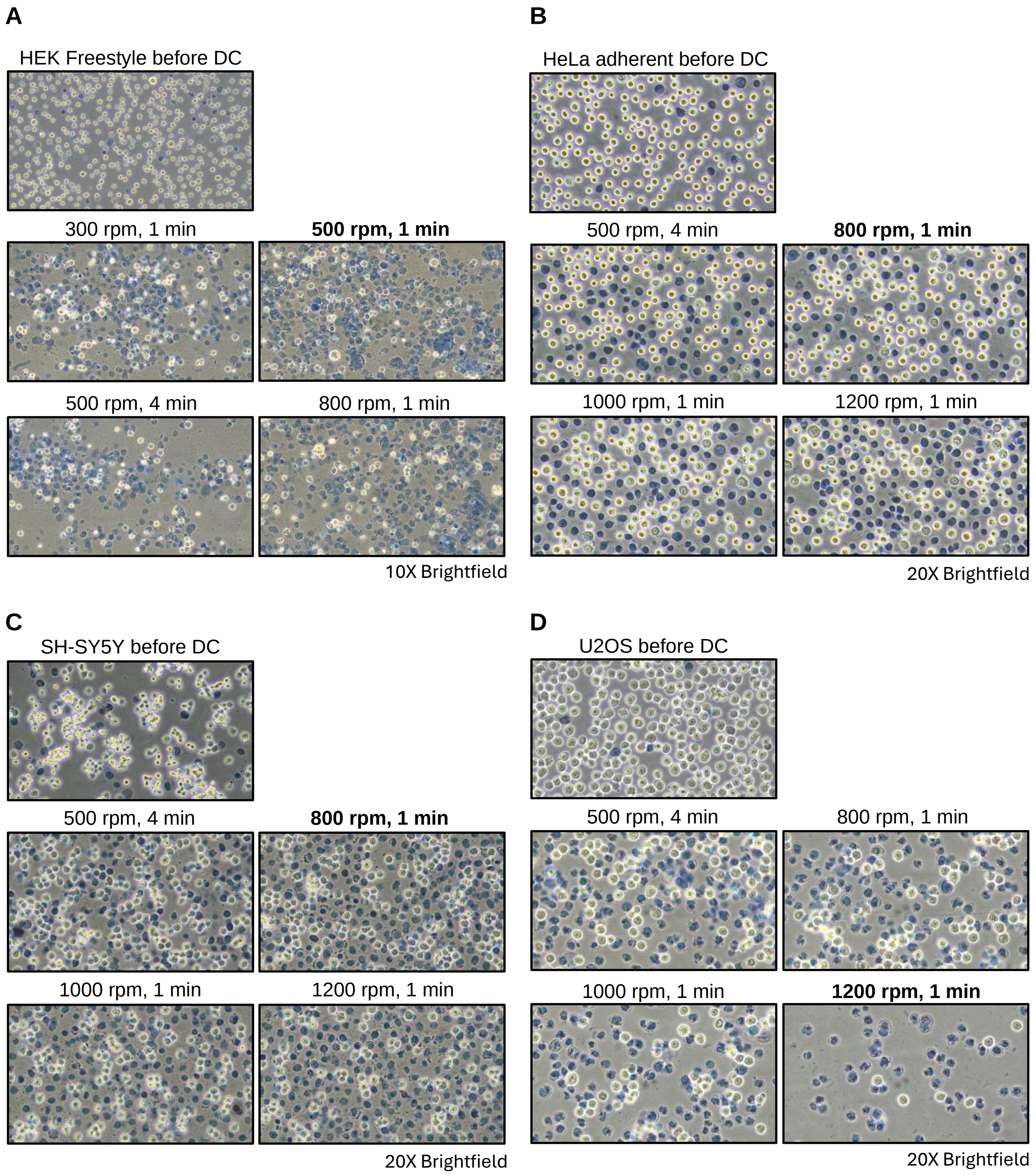

Supplementary Figure 3| Cell lysis efficiency of different cell lines by dual centrifugation
(A-D) Examination of cell integrity of HEK FreeStyle, HeLa adherent, SH-SY5Y and U2OS cells after dual centrifugation assessed by Trypan Blue staining. The corresponding Renilla luciferase assays of the resulting lysates are shown in Figure 3A-D. The DC conditions used are indicated above each image, and the condition with the best translation efficiency according to Figure A-D is indicated in bold letters.
